# Bispecific Radioligands (BRLs): Two Is Better Than One

**DOI:** 10.3390/jcm14165628

**Published:** 2025-08-08

**Authors:** Valeria Bentivoglio, Enrico D’Ippolito, Pallavi Nayak, Anna Giorgio, Chiara Lauri

**Affiliations:** 1 Nuclear Medicine Unit, Department of Medical-Surgical Sciences and Translational Medicine, “Sapienza” University of Rome, 00185 Rome, Italy; dippolito.1634666@studenti.uniroma1.it (E.D.); pallavi.nayak@uniroma1.it (P.N.); anna.giorgio@phd.unipd.it (A.G.); chiara.lauri@uniroma1.it (C.L.); 2 Department of Clinical and Molecular Medicine, “Sapienza” University of Rome, 00185 Rome, Italy

**Keywords:** radiopharmaceuticals, dual-target, nuclear medicine, tumor heterogeneity, imaging, tumor microenvironment

## Abstract

Inter- or intra-tumor heterogeneity refers to the genetic, epigenetic, and phenotypic variability that characterizes tumor cells. Regardless of its nature, this complexity represents a great challenge for diagnosis and treatment, since cells with different characteristics may respond differently to therapies, resulting in drug resistance and/or relapses. Furthermore, it has emerged that this heterogeneity can change over time or following a stimulus, such as a treatment. Molecular imaging is an essential tool in oncology. By enabling the identification of specific metabolic or receptor alterations of tumor cells, it provides a more accurate diagnosis, prognosis, and allows personalizing treatments. To date, addressing these challenges may be crucial for accurate diagnosis and therapeutic choice. In recent years, the design, synthesis, and characterization of bispecific radioligands (BRLs) with dual targeting capacity has emerged. In this review, we discuss the in vitro and in vivo studies of this new class of radiopharmaceuticals conducted so far, while assessing their potential advantages over traditional single-target radiopharmaceuticals.

## 1. Introduction

Significant challenges arise in the field of oncology due to both intra- and inter-tumoral heterogeneity. Intra-tumoral heterogeneity deals with several genetic and phenotypic characteristics within the same tumor cells, while inter-tumoral heterogeneity reflects the variability of the same histotype in different patients [1]. Every tumor has its distinct characteristics, and these traits may undergo changes over time either through natural progression or as a response to various factors, like treatments. This heterogeneity has impacts in real life medical settings by making treatments less effective due to the emergence of resistant cell populations during therapy that decrease the treatments effectiveness over time. It also complicates diagnosis and monitoring, as intra-tumoral variability may require more sophisticated diagnostic tools to accurately monitor tumor progression. It would be crucial to develop personalized treatment plans that specifically address the unique characteristics of each lesion and their dynamic changes over time, nevertheless this poses significant challenges. In particular, it would be desirable to have molecular imaging tools able to simultaneously bind multiple targets within the same tumor, in order to effectively capture intra- and inter-tumoral heterogeneity and to deeply explore the complex interactions within tumor microenvironment (TME). These involve cancer cells, tumor associated fibroblasts (known as TAFs or CAFto, which contribute in tumor progression and extracellular matrix remodeling, endothelial cells, which promote angiogenesis, and immune system, particularly tumor associated macrophages (TAMs), B and T lymphocytes as well as dendritic cells and natural killer cells (NKs), play a crucial role in this heterogeneity and in cancer progression [2]. Furthermore, soluble factors such as cytokines, chemokines, and growth factors (e.g., vascular endothelial factor –VEGF-, transforming growth factor beta -TGF beta-, interleukins) are crucial in regulating cancer-related inflammation and influencing immune responses [3,4,5].

To date, a wide range of novel radiopharmaceuticals have been developed, including small molecules, peptides and peptidomimetics, proteins, as well as full-length antibodies and antibody fragments, and have been used for the in vivo detection of tumors overexpressing one or more clinically relevant receptors through single photon emission computed tomography (SPECT) and positron emission tomography (PET) [6].

Unfortunately, since each tumor is different from others due to its heterogeneity, we would need a plethora of radiopharmaceuticals to target all possible biomarkers. Therefore, in recent years, a novel strategy has emerged to overcome these limitations allowing the simultaneous visualization of two biomarkers. This new class of radiopharmaceuticals is called radiolabeled heterodimers or bispecific radioligands (BRLs).

**Figure 1:**
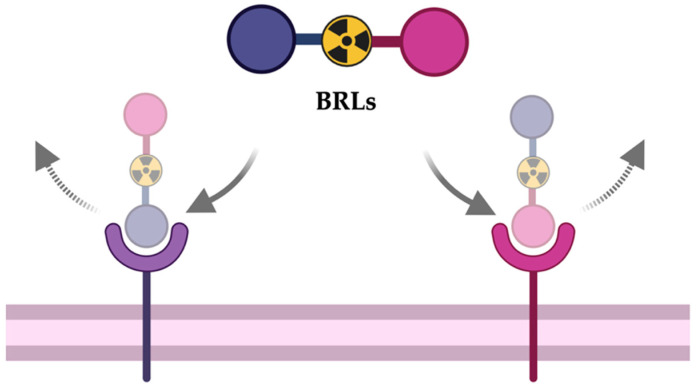
Schematic representation of a bispecific radioligand and the two specific receptors.

To date, almost all BRLs are composed of two peptide sequences, linked by a spacer, which includes the addition of a chelator useful for the coordination of a specific radionuclide (Figure 1).

Heterodimer peptides targeting more than one receptor can offer significant advantages over single-target tracers, for both diagnosis and therapy of tumors that simultaneously co-express more than one receptor type. As previously mentioned, heterogeneous receptor expression, in terms of density or distribution and type of receptor, may influence tumor progression and reduce targeted therapy efficacy; therefore, increasing the probability to target multiple receptors by means of BRLs would be crucial for therapy decision making [7].

In this review, we evaluate the in vitro and in vivo studies performed so far on this innovative class of radiopharmaceuticals, assessing their potential advantages over traditional single-target approaches.

## 2. BRLs for SPECT Use

To date, radiolabeled peptides have been extensively studied for nuclear medicine applications. Particularly, the use of short-lived isotopes, like ^99m^Tc and ^111^In, and the rapid biodistribution of peptides makes them excellent candidates for SPECT imaging applications. In this context, peptides containing Arginylglycylaspartic acid (RGD) sequence are extensively studied due to their high selectivity for integrins [8]. In particular, the selective targeting of the αVβ3 integrin has been largely investigated due its role in tumor angiogenesis [9]. In the same context, the bombesin (BBN) peptide, which targets gastrin-releasing peptide receptors (GRPR)—receptor overexpressed in different tumors—has also been widely studied [10,11,12].

### 2.1. Radiolabelling with Technetium-99-Metastable

Several BRLs have been labeled with Technetium-99m for SPECT imaging of different cancer histotypes, such as breast and prostate cancers, melanoma, glioma, and lung cancer.

In breast cancer, Ferreira and coworkers developed a novel dual-receptor heterodimeric peptide probe, ^99m^Tc-HYNIC-cRGDfk-NPY, composed by a RGD sequence, which targets tumor angiogenesis by recognizing integrin αVβ3, and a neuropeptide Y (NPY) receptor, which is overexpressed in 90% of human breast tumors. Its in vivo biodistribution in healthy mice demonstrated rapid systemic clearance, primarily via renal excretion, with secondary hepatobiliary elimination, in perfect agreement with its hydrophilic nature and increased gastric, bowel, and spleen uptake due to both low radiochemical purity and for the natural presence of NPY receptor in these tissues. The study also assessed the targeting of ^99m^Tc-BRL with two breast cancer cell lines xenografts: estrogen receptor-positive (MCF-7, expressing both NPY and αVβ3 receptors) and triple-negative (MDA-MB231, expressing high in αVβ3 and low in NPY). The results demonstrated high specificity and affinity for targeting in both cell lines, enhanced tumor uptake, which significantly decreased in the presence of an excess of unlabeled peptide (competitive targeting), and favorable tumor-to-muscle ratios; thus, this highlights its potential as a breast tumor imaging agent [13].

Other groups developed and characterized ^99m^Tc-labeled BBN-folate conjugates (^99m^Tc-BBN-folate) aiming at combining the targeting capabilities of BBN, directed toward GRPR, and folate, which targets folate receptors (such as FRα) commonly overexpressed in breast cancer cells. In vitro and in vivo evaluations demonstrated enhanced tumor uptake and retention of the radiopharmaceutical in T47D cells and tumors, which was strongly inhibited by the pre-incubation with cold folic acid or cold BBN, compared to monofunctional analogs. These findings suggest that ^99m^Tc-BBN-folate is another promising candidate for dual-receptor-targeted breast cancer imaging [14].

Afterwards, the diagnostic performance of ^99m^Tc-RGD-BBN for SPECT/CT imaging of integrin αVβ3 and GRPR, both commonly overexpressed in breast malignancies, was investigated by Ji and coworkers in 2015 in a clinical setting. Compared to conventional ultrasounds (US), ^99m^Tc-RGD-BBN (^99m^Tc-HYNIC-Glu-c(RGDyK)-BBN) SPECT/CT demonstrated higher sensitivity and negative predictive value (NPV) for primary breast tumor, axillary lymph nodes, and metastatic lesions. Conversely, specificity and positive predictive value (PPV) of the two methods were similar, thus suggesting that ^99m^Tc-RGD-BBN may offer significant advantages over standard imaging techniques in the evaluation of breast cancer. In particular, it can be used as an additional imaging tool eliminating the necessity for surgical biopsy and histopathologic examination [15].

In their 2009 study, Santos-Cuevas et al. proposed a novel hybrid radiopharmaceutical for imaging of prostate and breast cancers, namely ^99m^Tc-Tat-BBN (^99m^Tc-N2S2-Tat(49–57)-Lys^3^-BBN), which combines targeting properties of BBN with the HIV Tat-derived peptide (Tat(49–57)), a well-characterized cell-penetrating sequence designed to enhance cellular uptake and potentially facilitate nuclear localization. In vitro assays were conducted using prostate (PC-3) and breast (MCF-7, and MDA-MB-231) cancer cell lines, all known to express GRPR. The maximum uptake was presented in PC-3 at 4 h, in MCF7 at 2 h and MDA-MB231 at 5 min, dependent only on the different GRPR cell expression. The hybrid compound demonstrated significantly higher internalization rates compared to the control with only BBN, highlighting the functional contribution of the Tat sequence in promoting cellular penetration. Specific binding to GRPR was confirmed in all tested lines. In vivo biodistribution studies in PC-3 tumor-bearing mice revealed a tumor-to-muscle uptake ratio of 8.5 for the hybrid tracer, compared to 7.0 for the standard BBN tracer, suggesting improved tumor targeting and imaging contrast. These findings suggest that ^99m^Tc-Tat-BBN may allow SPECT imaging of different GRPR-expressing tumors, thus being particularly helpful in clinical practice [16].

Finally, in prostate cancer (PCa), ^99m^Tc-labeled iron oxide nanoparticles functionalized with prostate-specific membrane antigen (PSMA) and BBN showed high in vitro stability and enhanced affinity and specificity for PSMA- and GRPR-positive PCa cells. In vitro studies demonstrated efficient cellular uptake and receptor-mediated internalization, while preliminary imaging data supported its potential utility in imaging of PCa [17].

In 2012, Liu and coworkers developed another dual-targeting radiotracer, ^99m^Tc-RGD-BBN (^99m^Tc-HYNIC-Glu-c(RGDyK)-BBN), designed for SPECT/CT imaging of lung carcinoma in small-animal models, given the overexpression of both RGD and BBN in lung tumors. The radiolabeled compound exhibited high radiochemical purity, stability, and favorable pharmacokinetics. Planar and SPECT/CT images were acquired in mice bearing Lewis lung carcinoma (LLC) or bearing both inflammation and LLC. The high specific binding to tumor cells expressing the target receptors observed at in vitro assays was confirmed in vivo by the significant and specific tumor uptake, good contrast, and minimal background signal at SPECT/CT images. ^99m^Tc-RGD-BBN uptake in LLC xenografts was significantly higher compared to the uptake observed in inflammation; thus it potentially overcomes the limitations of ^18^F-FDG in distinguishing lung cancer from inflammation [18].

In 2016, de Oliveira et al. evaluated the potential of the heterodimer RGD-GX1 (^99m^Tc-HYNIC-E-[c-(RGDfk)-c(GX1)]), as novel radiotracer designed to bind both integrin αVβ3 (RGD) and tumor vasculature-specific markers, like α3β1 (GX1) for imaging tumor angiogenesis in experimental glioma models. The radiolabeled compound showed favorable biodistribution, good image quality at 1 h post injection (p.i.) and a higher tumor accumulation and retention of the radiolabeled BRLs, compared to ^99m^Tc-HYNIC-PEG4-c(GX1) [19].

In 2009, Yang and coworkers investigated a novel hybrid peptide, RGD-Lys-(Arg11)CCMSH, designed to combine the RGD motif with a modified alpha-melanocyte-stimulating hormone (α-MSH) analog, which targets the melanocortin-1 (MC1) receptor overexpressed in melanoma cells. The hybrid peptide demonstrated a strong binding affinity to the MC1 receptor. In vivo studies in B16/F1 melanoma-bearing mice revealed high tumor uptake and sustained retention, with minimal accumulation in non-target tissues, except for the kidneys. SPECT/CT imaging successfully visualized melanoma tumors using the radiolabeled peptide, highlighting its potential as a diagnostic tool. A single 3 h treatment with the hybrid peptide resulted in a significant 65% reduction in clonogenic survival of B16/F1 melanoma cells compared to untreated controls, suggesting also its therapeutic efficacy. However, further optimization is needed to reduce renal uptake, potentially through amino acid co-injection strategies or structural modifications, to enhance its suitability for clinical applications [20].

### 2.2. Radiolabelling with Indium-111

Most of the available studies on ^111^In-labeled BRLs predominantly target PCa.

In 2021, Bandari et al. used dual-targeting small-molecule/peptide radiotracers able to bind both GRPR and PSMA. They synthesized an ^111^In radiolabeled peptide ([^111^In]–DO3A), incorporating DUPA (PSMA ligand) to a BBN antagonist (BBN ANT) targeting GRPR. Using competitive binding assays on PC-3 (Prostate cancer cell line GRPR+) cells and LNCaP membranes (Prostate cancer cell line PSMA+), this compound showed strong and high binding affinity to their respective targets. Biodistribution studies in murine models showed favorable pharmacokinetics, with tumor uptake in PC-3 tumor-bearing mice within 1 h. Micro-SPECT scans in xenografted mice at 4 h p.i. revealed high tumor accumulation and retention, and low off-target uptake of the radiopharmaceutical [21].

A similar approach was adopted in 2019 by Mitran et al. by using heterodimer composed of two key binding elements: a Glu-Ureido-based PSMA inhibitor DUPA and BBN-based antagonist RM26 for GRPR targeting. These two components were linked through a flexible polyethylene glycol (PEG_6_) spacer and coordinated via a NOTA chelator for radiolabeling with either ^68^Ga and ^111^In for PET and SPECT applications, respectively. The compound NOTA-DUPA-RM26 was synthesized and successfully radiolabeled with ^111^In with high radiochemical purity and stability. In vitro binding assays demonstrated that the heterodimer retained high affinity for GRPR, while exhibiting moderate affinity for PSMA. Importantly, competitive binding studies confirmed the specificity of the compound for both targets. Moreover, biodistribution studies on mice bearing PC3-PIP xenografts showed rapid renal clearance with high tumor/background (T/B) ratios, supporting a favorable pharmacokinetic profile. Blocking experiments using excess unlabeled ligand significantly reduced tumor uptake, thus confirming the high dual-targeting specificity [22].

Stott Reynolds et al. evaluated a hetero-bivalent radiotracer designed to target GRPR and the integrin αVβ3, in PC-3, DU-145, and LNCaP cell lines. The researchers synthesized a compound that incorporates a BBN-derived GRPR antagonist (RM2) and a cyclic RGD peptide targeting integrin αVβ3, linked via a DOTA chelator, enabling radiolabeling with both diagnostic (^111^In) and therapeutic radionuclides (^177^Lu). In particular, [RGD-Glu-[^111^In-DO3A]-6-Ahx-RM2] showed favorable pharmacokinetic specific binding to both receptors, with favorable affinity profiles. Cell-based assays in GRPR-positive (PC-3) and integrin-expressing (U87-MG) cell lines supported the high performance of the bispecific nature of the agent. Micro-SPECT/CT images at 20 h p.i. showed high-quality, high-contrast, whole-body images with minimal uptake in non-tumor tissue, especially the liver and kidneys [23]. Moreover, the possibility to replace ^111^In with ^177^Lu would open new possibilities for theragnostic approaches.

^111^In-labeled BRLs have also been used in neuroendocrine tumors overexpressing somatostatin receptor subtype 2 (SSTR2). Capello and colleagues, in 2006, explored a novel therapeutic strategy using RGD-^111^In-DTPA-octreotate that combines RGD-containing peptide with somatostatin analogs, targeting SSTR 2. This heterodimer shows tumoricidal effect, with respect to the two radiolabeled monopeptides. RGD-^111^In-DTPA-octreotate showed high uptake and retention in the implanted pancreatic CA20948 tumor rat. In vitro, the unlabeled peptide RGD-DTPA-octreotate induced a significant increase in caspase-3 levels in various cell lines in comparison with single peptides. A major drawback was its high renal uptake, thus limiting its use for radionuclide therapy [24].

Another group evaluated a novel hybrid radiopharmaceutical, RGD DTPA Tyr^3^ octreotate, designed for receptor-targeted radionuclide therapy of tumors overexpressing SSTR2. This multifunctional peptide combines the somatostatin analog Tyr^3^ octreotate, which confers high affinity for SSTR2, with an apoptosis-inducing RGD motif, and the chelator DTPA to enable ^111^In radiolabeling. In vitro experiments on SSTR2-positive CA20948 and other tumor cell lines demonstrated rapid and receptor-specific uptake of the radiolabeled compound. The internalization was effectively blocked by an excess of octreotide, but not by RGD alone. In vivo biodistribution studies in CA20948 tumor-bearing mice confirmed high tumor uptake and retention comparable to that of DOTA Tyr^3^ octreotate. However, high renal accumulation was identified as a potential drawback. Further in vitro investigations highlighted the pro-apoptotic potency of the unlabeled RGD DTPA octreotate peptide. It induced significantly greater caspase-3 activation and tumoricidal effects than monopeptides alone. Nonetheless, high renal retention of the radiolabeled hybrid suggests limitations for therapeutic application, despite promising antitumor activity. Further optimization, particularly to mitigate renal uptake, is needed to fully realize its therapeutic potential [25].

Overall, these studies highlight the potential of the ^111^In dual-receptor targeting for improving the diagnostic accuracy, in particular in PCa, due to the variability of the receptor expression seen among patients. The bispecific approach offers the advantage over monovalent radiopharmaceuticals by increasing the chance of tumor detection, decreasing FN results and avoiding multiple diagnostic imaging procedures. Nevertheless, compared to 99m-Tc, ^111^In is burdened with its longer half-life, poorer image quality, and less favorable dosimetry and higher costs.

### 2.3. Radiolabelling with Iodine-125

Very few papers were published on iodine-125 labeled BRLs.

Kim et al. developed a novel iodine-125 radiolabeled radiopharmaceutical for glioma imaging. The compound combines cMBP, a mesenchymal–epithelial transition factor (c-Met)-binding peptides and a cRGDyk peptide, which are both often overexpressed in tumor cells and newly formed vessels. Once combined the two peptides using “click” chemistry (a bio-orthogonal reaction), the polypeptide was radiolabeled using Chloramine-T to create the bifunctional imaging agent ^125^I-cMBP-click-cRGDyk. The results showed high affinity and specificity for the target receptors and in mouse models of glioma, the tracer selectively accumulated in the tumor with better results compared to single ligands; although, a further optimization of the BRL to avoid the high renal clearance and the decreasing tumoral uptake over time is needed to be translated in clinical practice [26].

Ayman et al. synthesized several compounds targeting PSMA and GRPR, which were then analyzed in vitro on PC-3PIP cells and in vivo in mice bearing PC-3 and LNCaP xenografts. Only the radioiodinated heterodimer ^125^I-BO530, which combines a PSMA-targeting urea-based inhibitor with a GRPR antagonist (RM26), linked via a PEG_2_ linker, demonstrated high affinity for both receptors and high tumor uptake (~30–35% ID/g at 3 h), which was sustained up to 24 h. The low uptake in healthy organs suggests a favorable theranostic profile, potentially by labeling it with therapeutic isotopes [27].

These studies demonstrated how the integration of bispecific peptides radiolabeled with iodine 125 can improve the selectivity and efficacy of tumor imaging, but further preclinical studies are needed to assess the potential clinical role in diagnosis and precision therapy.

Overall, despite all the above-mentioned studies adopting different strategies to bind different targets in several tumors, these findings underline the importance of BRLs for dual-receptor targeting in many clinical scenarios aiming at reducing FN results and at avoiding multiple diagnostic imaging procedures. Nevertheless, several optimizations should be performed to reduce renal uptake. Last, but not least, the modular design of the heterodimer could be modified also for theranostic purposes, by replacing the imaging radionuclide with therapeutic isotope. Several SPECT isotopes have been proposed for the radiolabeling, with technetium-99m being the best candidate due to its wider availability, lower cost, favorable dosimetry, and better biodistribution compared to ^111^In and ^125^I.

Radiolabeled BRLs for SPECT use are summarized in Table 1.

## 3. BRLs for PET Use

PET has become an essential tool in clinical oncology for detailed imaging of molecular targets in the body with higher precision and accuracy than SPECT imaging. Therefore, several efforts are currently made to develop new PET radiopharmaceuticals able to target two molecular sites simultaneously within the TME, thus overcoming the lower sensitivity and specificity of single target tracers and the limitations of SPECT imaging [28]. Recent progress in radiochemistry, together with novel strategies such as click chemistry [29], SiFA-based ligation [28], or prosthetic group strategies [30,31,32,33], led to the development of BRLs labeled with fluorine-18 (^18^F), Gallium-68 (^68^Ga), or Copper-64 (^64^Cu). These advancements allow for automated manufacturing and clinical application of BRLs compounds for dual target PET imaging in different cancer types including glioblastoma, melanoma, PCa, breast cancer, and hepatocellular carcinoma.

### 3.1. Radiolabelling with Fluorine-18

^18^F is particularly used in clinical practice given its favorable physical characteristics, such as a relatively short half-life (t1/2 = 109.7 min), low positron kinetic energy (Eβ+ = 0.635 MeV), and its widespread availability and suitability with automated synthesis modules. ^18^F-BRLs have shown some clinical potential primarily due to the progress in radiochemistry, including the use of prosthetic groups ([^18^F]SFB, [^18^F]fluorobenzoyl), as well as click-chemistry-based (strain-promoted azide-alkyne cycloaddition, SPAAC) methods which provided mild site-specific and efficient labeling while retaining biological activity.

Cheng et al. reported a BRL for both the integrin αvβ3 and MC1 receptors, based on SiFA linkage, to diagnose melanomas. The SiFAlin-moiety exhibited a stable positive charge over time and allowed binding ^18^F in one step. The ex vivo studies showed higher tumor uptakes compared to the background [28]. Meanwhile, Chen et al. synthetized a BRL for the diagnosing hepatocellular carcinoma (HCC). The radiopharmaceutical was prepared by conjugating TJ12P2, a peptide against Glypican-3 (GPC3), expressed in 95% of HCC but rarely present in healthy liver and 2-PMPA, a potent PSMA inhibitor via a PEG linker obtaining the polypeptide T2P. T2P was then radiolabeled with ^18^F using AlCl3 with a high radiochemical purity (97.86 ± 0.70%). PET images acquired 30 min p.i. showed tumor uptake and tumor-to-muscle (T/M) ratios comparable to those obtained by single GPC3-targeted radiopharmaceutical [34].

Wu et al. developed a dual-specific peptide for gliomas imaging with RGD and ATWLPPR for Neuropilin-1 (NRP-1) through a glutamate linker and labeled by reacting the [^18^F]fluoride–aluminum complex with the NOTA chelator. The labeling efficiency (LE) ranged between 30 and 40% and the radiochemical purity reached 95%. The uptake of the radiopharmaceutical was substantial in U87MG cells (Glioblastoma cells line). Especially in the later points, at 120 min, the uptake of the BRL was 10.02 ± 0.90% vs. 8.75 ± 0.77% and 5.29 ± 0.81% of the monomeric counterparts, [^18^F]Fal-NOTA-RGD, [^18^F]FAl-NOTA-ATWLPPR. In tumor targeting experiments using U87MG glioblastoma-bearing mice, the BRL showed enhanced retention as compared to monomeric analogs. Dual receptor targeting provided better sensitivity, particularly for gliomas co-expressing both angiogenesis-related targets [31]. Also, RGD-A7R heterodimer, radiolabeled with ^18^F-SFB solution for dual targeting of αvβ3 and VEGFR2 was conceptualized for imaging angiogenic biomarkers. The radiopharmaceutical showed good tumor uptake and receptor specificity in U87MG xenografts, which provided the proof of concept for simultaneous targeting of angiogenic markers in different signaling axes. The radiopharmaceutical showed a non-specific uptake in stomach and bowel although is mainly excreted through the renal-urinary route, as suggested by the kidney’s uptake at early time points. Therefore, innovative strategies are needed to improve the kinetics [32]. Ma et al. disclosed the development of a bispecific NT-PSMA BRL targeting both internal and external tumoral heterogeneity through the dual recognition of neurotensin receptor (NTR1) and PSMA. The peptides, Glu-urea-lys(Ahx) for PSMA and NT20.3 for NTR1 were linked through N-(Azido-PEG3)-N-bis(PEG3-NHS ester), a trifunctional linker. The binding assay using PC-3 cells showed a IC50 value of 50.2 nM where the NTR1 targeting peptide retained its property compared with the corresponding monomer, whereas the PSMA targeting peptide showed reduced binding affinity. The in vivo studies confirmed these results; indeed, the BRL uptake in LnCaP tumor models (PSMA positive/NTR1 negative) was lower than PSMA monomer. Authors suggested that the introduction of a relatively large NT peptide motif may influence the binding affinity [29].

Chen et al. described a pharmaco-modulated library of fibroblast activation protein inhibitors (FAPI)/biotin, labeled with ^18^F which take advantage of the FAP-binding affinity of quinoline-based moieties and the pharmacokinetic modulation by biotin (permeability, solubility, and clearance). In particular, [^18^F]AlF-NSFBP4 in in vivo preclinical setting demonstrated specific and high accumulation of the BRL in HT1080-FAP tumor-bearing mice up to 4 h p.i., thus making it a good candidate for delayed scans. This design provides an example of how to rationally incorporate modular systems (e.g., biotin) into a hetero-bivalent platform to enhance imaging performance [35].

Several studies investigated ^18^F-labeled BRLs consisting of RGD peptides and BBN analogs. Liu et al. labeled integrin αvβ3/GRPR dual receptor-targeted BRLs with ^18^F, ^64^Cu, and ^68^Ga for PET visualization of breast cancer. These radiopharmaceuticals demonstrated high binding affinities, rapid blood clearance, and high T/B ratios. Particularly, the ^18^F-equipped variant was tested on T47D and MDA-MB-435 tumor cells (human breast cancer cell lines) showing higher uptake on the T47D cells than on the MDA-MB-435 tumor cells, maybe due to the higher GRPR expression of the T47D cells. The micro-PET images in T47D and MDA-MB-435 tumor-bearing mice showed prominent uptake in the kidneys and urinary bladder at early time points, due to renal excretion, a rapid plasma clearance and a lower background compared to the other BRLs labeled with ^64^Cu, and ^68^Ga [30]. To further explore a similar strategy, the same group developed a PEGylated ^18^F-labeled RGD-BBN BRL (^18^F-FB-PEG3-Glu-RGD-BBN) that increased hydrophilicity of the compound, while exhibiting improved in vivo pharmacokinetics. This conjugate showed better tumor accumulation (6.35 ± 2.52, 4.41 ± 0.71, and 2.47 ± 0.81% ID/g at 30, 60, and 120 min). The rapid clearance of the radiopharmaceutical from normal organs and the tumor/non-tumor (T/NT) ratio increased with time [36]. Yan et al. perpetuated the heterodimer model by adding AEADP, a symmetric linker, composed of two carboxylic acid functional groups, used to link RGD and BBN peptides by amide bonds with the free amino groups of the peptides, and one free amino group used for ^18^F labeling through SN2 nucleophilic reaction with ^18^F-SFB. Results showed that this linker did not affect the biological activity of the peptides [37]. Li et al. described one of the first ^18^F-labeled RGD-BBN dimers for PET imaging of PCa. This radiopharmaceutical covalently links an RGD motif and a BBN analog via Boc-Glu(OSu)2, obtaining different formulations, Boc-Glu-BBN-RGD with RGD on the sidechain δ-position and Boc-Glu-RGD-BBN with BBN on the sidechain δ-position. The authors observed no differences between the two formulations, deciding that the final product can be a mixture of two compounds. The complex exhibited good receptor-binding affinities to two receptors using PC-3 cell lines and micro-PET scans performed on a PC-3 xenograft model showed higher T/B ratios than the monomeric analogs [33].

Taken together, the results of these studies demonstrate the promising utility of ^18^F-labeled RGD-BBN heterodimers as a class of dual-targeting PET radiopharmaceuticals. The dual targeting of integrin αvβ3 and GRPR results in synergistic tumor targeting and detection, especially in cancers, such as prostate and breast cancers, that are characterized by co-expression of these receptors. More recently, Liu et al. extended the concept of the hetero-dimeric approach by developing FAPI-RGD, targeting both FAP in tumor stroma and integrin αvβ3. Two versions of the heterodimer were analyzed in this study, one conjugated to ^68^Ga, ([^68^Ga]Ga-LNC1007) and the other to [^18^F]AlF ([^18^F]AlF-LNC1007). Stability test results showed that both BRLs remained intact after incubation with fetal bovine serum (FBS) in PBS for 2 h. In FAP- and integrin-positive tumor models, both BRLs exhibited high tumor uptakes. However, the ^18^F-labeled BRL was tested in six patients with malignant tumors. The scans revealed that the lesions could be clearly visualized from 5 min p.i. and retained a favorable contrast until 120 and 180 min p.i., suggesting a favorable imaging potential [38].

In conclusion, fluorine-18-labeled BRLs represent a promising class of PET radiopharmaceuticals, thanks to the excellent properties of ^18^F and the continuous advances in radiolabeling techniques. Dual targeting strategies, particularly towards angiogenic and tumor-specific receptors, have demonstrated effective tumor localization, increased retention, and improved image contrast. These preclinical and clinical results highlight the potential of ^18^F-labeled hetero-dimeric BRLs for more sensitive and specific oncology imaging.

### 3.2. Radiolabelling with Gallium-68

As previously shown, BRLs directed against PSMA and GRPR might overcome limitations of standard molecular imaging of PCa by assessing intra-tumoral heterogeneity of different receptor expressions.

In one of the first papers published in this field, Eder et al. investigated a small molecular weight BRL, composed of nonapeptide BZH3 which targeted GRPR and the urea-based PSMA inhibitor Glu-urea-Lys(Ahx)-HBED-CC. Results showed a strong binding affinity on LNCaP and PC-3 cells, with a GRPR specificity nearly identical to that of the monomeric analog; however, the affinity of the PSMA specificity was reduced by a factor of 2.3. Preclinical studies on LNCaP and PC-3 xenografts showed favorable pharmacokinetics with a humble uptake of ^68^Ga-labeled compound in healthy pancreas tissue. However, it should not raise clinical problems since prostate tumors usually do not metastasize in pancreas [39]. This study established a fundamental background for the further development of bispecific imaging agents.

Afterward, Liolios et al. described a novel structural family of PSMA/GRPR-targeting radioligands, which were optimized to obtain better pharmacokinetic profiles. Indeed, the modification of linker chemistry and molecular design resulted in a faster clearance from non-target tissues and, thus, a better T/B ratio. In particular, the design was based on H2N-PEG2-[D-Tyr6β-Ala11,Thi13,Nle14]BBN(6–14), a BBN analog, and on Glu-ureido-Lys, a urea-based inhibitor of PSMA. The two peptides were coupled through copper(I)-catalyzed azide–alkyne cyclo-addition to the bis(tetrafluorophenyl)ester of the chelating agent HBED-CC, via amino acid linkers. The radiolabeling with Gallium-68 provided 99% of radiochemical purity and a high stability over time, using LNCaP and PC-3 cells. Results showed maximal cellular uptake at a similar rate between monomers and BRL after 20 min for PC-3 cells and 40 min for LNCaP cells. The ligands showed good affinity for both receptors and (on both αvβ3 and PSMA) displayed better imaging capabilities than their radioiodinated counterparts, consolidating the use of ^68^Ga-BRLs in PCa diagnosis. Moreover, the introduction of the HE spacer resulted in an optimization of the pharmacokinetics with a reduction in the kidney uptake [40].

Likewise, Mendoza-Figueroa et al. reported on the BRL trilogy ^68^Ga-iPSMA-BBN for PET imaging. This molecule was a hybrid molecule targeted PSMA, using the peptide Nal-Lys-CO-Glu-OH and GRPR, using Lys3-BBN. After radiolabeling with Gallium-68 and using DOTA as chelating agent, stability was tested in human serum at 37 °C for 3 h. Results showed less than 6% binding to the serum proteins. In vitro binding and in vivo biodistribution showed high tumor uptake with the expected favorable clearance that qualifies the agent as an alternative to single-target agents to clearly define heterogeneous tumors with varying receptor expression profiles, such as advanced or treatment-resistant PCa [41].

Mitran et al. developed a PSMA/GRPR heterodimer for PET and SPECT imaging. This compound was obtained by the conjugation of Glu-Ureido-based PSMA inhibitor DUPA and RM-26, which target GRPR coupled via a glutamic acid bearing NOTA chelating agent. The authors chose to use a lipophilic linker to have a better binding affinity to PSMA; nevertheless, despite the fact they observed rapid blood clearance, high tumor uptake, and low uptake in normal tissues and excretory organs in preclinical studies, binding experiments showed a relatively low affinity [22].

More recently, Liolios et al. described the development of a BRL that combines PSMA-617, which has been already approved for clinical use as a PSMA-targeting prostate tumor agent, with RM2, a binding peptide of GRPR. The bispecific agent demonstrated strong receptor binding, good biodistribution, and solid tumor localization in animal models with a tumor/tissue ratios increased for most of the tissues over time. The low off-target organ radioactivity makes this BRL suitable for further studies suggesting its potential for clinical application in imaging and therapy [42]. These radiopharmaceuticals represent a unique example of conjugation with a GRPR antagonist instead of an agonist, to increase safety and receptor affinity.

Together, these studies highlight the considerable advances made in the development of BRLs targeting both PSMA and GRPR. These agents provide an elegant solution to the problem of receptor heterogeneity in PCa, thus contributing to the (r)evolution towards personalized nuclear medicine. Additional clinical investigation is required; however, the preclinical data are highly compelling for the clinical translation of these dual-targeted probes for improved diagnosis, patient stratification, and perhaps theranostic use.

As previously mentioned, RGD-BBN-based heterodimers which can dual-target integrin αvβ3 and GRPR have shown great potential in solid tumor molecular imaging, especially for breast cancer and PCa models, by combining tumor vascular targeting specificity of RGD peptides (binding to integrin αvβ3) with the tumor-cell receptor targeting affinity of BBN analogs (binding to GRPR).

In a comparative study on ^68^Ga-NOTA-RGD-BBN, ^18^F and ^64^Cu-labeled RGD-BBN radiotracers in MDA-MB-435 breast cancer xenografts, the authors reported better biodistribution of ^68^Ga-labeled BRLs compared to the others and good pharmacokinetics (tumor uptake of ^68^Ga-NOTA-RGD-BBN was higher than that of the ^18^F and ^64^Cu-labeled RGD-BBN radiotracers) from 30 to 120 min p.i. despite a higher blood retention was observed [30].

In another study from the same research team, ^68^Ga-NOTA-RGD-BBN confirmed high affinities to both receptors and the selective tumor uptake, as well as fast normal organ clearance. This is consistent with the benefit of the heterodimer design to compete in the complex TME where angiogenesis and receptor overexpression co-localize [43].

Zhang and colleagues published the first clinical studies using ^68^Ga-BBN-RGD PET in patients with focal breast cancer or PCa. Well-defined tumor uptake with good contrast was observed along with satisfactory safety and pharmacokinetic profiles. In their first trial, 13 patients with PCa were enrolled. Results confirmed that ^68^Ga-BBN-RGD is specific for GRPR and integrin αvβ3, demonstrating the clinical feasibility of BRLs and provided evidence for a clinical trial in multispecific receptor-positive tumors. In another study on breast cancer, ^68^Ga-BBN-RGD PET/CT allowed the demonstration of GRPR and integrin αvβ3 co-expression in a substantial portion of tumors. Radiolabeled-BRL provided good T/B ratio in both primary tumors and metastases, with 8% of FP results. However, further larger scale clinical investigations are needed [44,45].

Finally, Amraee et al. performed human dosimetry calculations of ^68^Ga-NODAGA-RGD-BBN, based upon preclinical biodistribution, reporting favorable dosimetry safety profile; thus, potentially facilitating the translation into clinical trials [46].

Taken together, these studies highlight the utility of RGD-BBN in dual-receptor PET imaging, particularly for breast and prostate tumors. These agents improve diagnostic accuracy and have promise for staging and monitoring treatment response by targeting the molecular heterogeneity of tumors using αvβ3 and GRPR dual-inhibition. The strong translation to the clinic achieved in recent years strongly recommends the further clinical implementation of both targeted approaches in oncologic nuclear medicine [46].

Alternative strategies to exploring alternative receptors combinations have been proposed to provide a more comprehensive overview of the TME. In particular, there is a growing interest in co-targeting FAP with integrin αvβ3 for imaging of CAFs and tumor vasculature simultaneously.

Zang et al. recently described the development and preclinical assessment of ^68^Ga-FAPI-RGD. Preclinical data showed high and selective accumulation in Panc02 (FAP and integrin-expressing) xenografts, good pharmacokinetics, and rapid elimination from non-target tissues. Radiation dosimetry assessment showed promising safety profiles (the organ with the highest effective dose was the urinary bladder wall with 2.26 × 10^−1^ mSv/MBq). PET/CT studies in patients with lung cancer lesions showed that the uptake in cancer lesions could be clearly visualized at the early 15 min scan, which became clearer at 1 h and 2 h scans [47].

Another step towards clinical translation of this novel approach was achieved by Zhao et al., who successfully performed ^68^Ga-FAPI-RGD in 22 patients with different malignancies (pancreas, lung, and colorectal cancer). In this study, the authors evaluated the in vivo distribution pattern of the BRL and tumor uptake overtime using PET scans performed at 1 and 3 h p.i. PET imaging demonstrated increased high T/B ratios over time, with a SUVmax from the delayed scan (3 h) significantly higher than SUVmax from routine scans (1 h). In comparison with ^18^F-FDG PET, the SUVmax of primary tumors from ^68^Ga-FAPI-RGD PET/CT were significantly higher, particularly in non-small cell lung cancer and in esophageal, breast, and pancreatic cancers [48].

Taken together, these studies highlight the broad applicability and clinical potential of dual-targeting radiotracers for PET in which signals from tumor cells and their stromal microenvironment are combined. Whether through co-targeting FAP and integrin, as in FAPI-RGD or GRPR, and angiogenesis markers, as in BBN-RGD, these BRLs with their enhanced BD capability may be able to improve diagnostic accuracy and treatment patient selection. Ability to non-invasively image several hallmarks of cancer in one imaging approach presents a robust foundation for theranostics, particularly in tumors with heterogeneous biology and variable receptor expression.

In addition to double-targeted clinical PET radiopharmaceuticals that have been exploited so far, a second generation of BRLs would address alternative receptor-pairing combinations of the multitude of heterogeneous metabolic pathways expressed by different kinds of malignancy. These novel classes of BRLs include dual targeting to PSMA/FAP, GRPR/VPAC1, and EGFR/integrin αvβ3, as well as Glypican-3/PSMA, and others, and reflect the attempt of molecular imaging to be tailored to tumors’ heterogeneity and to specific microenvironmental niches.

Wang et al. used ^68^Ga-PSFA-01, the first BRL-containing PSMA- and FAP-targeting motifs for PET imaging. Preclinical studies showed rapid accumulation in PSMA- and FAP-positive tumors. In a first human study, PSFA-01 was successfully performed in one patient with PCa, obtaining enhanced detection sensitivity and exhibiting satisfactory imaging quality. It surpassed both ^68^Ga-PSMA and ^68^Ga-FAPI scans in terms of the number and SUVmax of metastatic lesions displayed. This dual-receptor approach widens the detection capability of PCa heterogeneity phenotypes and also shows potential in theranostics [49]. Concurrently, interest is increasing in the use of FAPI-based imaging for non-oncological purposes. Chen et al. employed ^68^Ga-FAPI-LM3 for the visualization of early pulmonary fibrosis, a non-malignant but fibroblast-abundant disorder. The data presented in this study underlined that the dual-targeting concept can also be transferred to fibrotic or inflammatory diseases, therefore further increasing clinical potentiality of FAPI-based BRLs beyond oncologic purposes [50].

Other groups examined GRPR tethering to other neuro-receptors. Bodin et al. developed ^68^Ga-labeled dual peptide heterodimers against NTS1 and GRPR. Such receptors tend to be co-expressed in some neuroendocrine tumors and PCa. These BRL showed high binding affinity and favorable imaging characteristics, indicating greater diagnostic specificity for dual neuro receptor-expressing tumors [51]. Similarly, Vall-Sagarra et al. reported hetero-bivalent ligands targeting both GRPR and NPY(Y1) receptors for breast cancer detection. Their investigations demonstrated that this combination resulted in increased binding in receptor-rich tumors and could potentially lead to signal specificity compared with single-receptor radiopharmaceuticals [52].

Lindner et al. also studied GRPR/VPAC1 heterodimers for PCa. The improvements over previous approaches were only modest, but the dual-targeting of two G-protein coupled receptors has potential for tumors with heterogeneous receptor expression [53,54].

Going beyond GPCRs, EGFR and integrin αvβ3 have also been included in dual-targeting constructs. A dual-targeted ^68^Ga-NOTA-RGD-GE11 heterodimer, which can simultaneously image integrin and EGFR expression targeting agents in preclinical tumor models. These approaches address the need to image aggressive tumors such as triple-negative breast cancer or EGFR-driven malignancies with improved accuracy [55,56].

Lastly, Ghanipour et al. proposed a Glypican-3/PSMA bispecific PET radiopharmaceutical, emphasizing the GPC3 as a promising candidate for liver cancer. This molecule allowed for bimodal imaging of hepatocellular carcinoma and PCa models—perhaps paving the way for wider theranostic application in which multiple cancer types could be targeted using a single imaging agent [34].

Wen et al. also promoted clinical translation by presenting preclinical and first-in-human data of ^68^Ga-LNC1015, a dual integrin αvβ3 and GRPR-targeting BRL. In this study, 11 patients with suspected breast cancer were enrolled. After a week, an ^18^F-FDG PET/CT was performed as a comparison. Results showed that the BRL is well tolerated. The kidneys were the main excretory organs due to the strong signal measured in the bladder. Additionally, the effective dose was lower than the dose limit required by the FDA. ^68^Ga-LNC1015 detects more micrometastatic lymph nodes that were not found by [^18^F]FDG PET/CT. This research validated that the bispecific radiopharmaceuticals could efficiently image breast tumors with a better contrast compared to its monovalent design, further validating the dual-targeting power in complicated tumor systems [57].

Taken together, these new dual-targeting strategies highlight an important transition in PET radiopharmaceuticals development: from single dominant marker targeting to bispecific agents that address tumor heterogeneity. When angiogenesis markers, tumor cell receptors, stromal signatures, and neuropeptides have been combined, these BRLs contribute to a broader understanding of tumor biology, thus offering new insights for personalized diagnosis and therapy.

### 3.3. Radiolabelling with Copper-64

Copper-64 is a well-suited PET radionuclide for labeling BRLs due to its relatively long half-life (12.7 h), which allows for extended imaging windows, and its stable coordination via chelating agents such as DOTA or NODAGA. In the context of dual-targeting agents—especially those directed at both integrin αvβ3 and ^64^Cu-GRPR—this approach has demonstrated favorable pharmacokinetics and strong tumor uptake, offering high-contrast imaging potential.

Liu et al. conducted a pioneering comparative evaluation of RGD-BBN BRL radiolabeled with ^18^F, ^64^Cu, and ^68^Ga for dual-targeted PET imaging of breast cancer, with the aim of exploiting the overexpression of αvβ3 integrins and GRPR in tumor neovasculature and cancer cells, respectively. The study demonstrated that ^64^Cu-DOTA-RGD-BBN displayed markedly superior in vivo performance compared to its ^18^F and ^68^Ga counterparts. Specifically, ^64^Cu-DOTA-RGD-BBN exhibited increased metabolic stability, prolonged blood, enhanced tumor retention, and significantly higher T/B ratios at late imaging time points. The prolonged half-life of ^64^Cu, combined with its favorable positron emission energy (β+, 17.4%), facilitates high-resolution imaging at delayed time points, offering improved lesion contrast and diagnostic reliability. Moreover, the DOTA chelating agent used in this construct provided robust complexation with ^64^Cu, reducing transchelation in vivo [30].

In other investigations, the same research group confirmed the enhanced tumor uptake of ^64^Cu-DOTA-RGD-BBN relative to its monomeric analogs—^64^Cu-DOTA-RGD and ^64^Cu-DOTA-BBN—thus demonstrating the added value of bispecificity in molecular imaging. The construct showed synergistic binding to αvβ3 and GRPR. This dual-receptor engagement led to increased lesion detectability, particularly in heterogeneous tumor models, where mono-receptor targeting would be insufficient. The PET imaging data also indicated rapid tumor accumulation and sustained retention, essential parameters for clinical imaging applications requiring high sensitivity and specificity [58].

Based on these findings, Jackson et al. developed a next-generation BRL, ^64^Cu-NO2A-RGD-Glu-6-Ahx-BBN(7–14)NH_2_, designed with an optimized molecular scaffold incorporating a flexible glutamic acid spacer and an aminohexanoic acid (Ahx) linker to modulate spatial orientation and receptor accessibility. The use of the NO2A chelator allowed for efficient and stable radiolabeling with ^64^Cu under mild conditions. In vivo PET imaging in PCa xenografts showed strong and specific uptake, predominantly renal clearance, and minimal hepatobiliary accumulation, addressing a common limitation of peptide-based tracers. The construct displayed nanomolar affinities for both αvβ and GRPR, and its favorable pharmacokinetic profile underlines its potential for clinical translation in the imaging of GRPR- and integrin-expressing tumors [59].

Lucente et al. proposed a trivalent hetero-trimeric design with the synthesis of ^64^Cu-RGD_2_-BBN, integrating two RGD sequences and one BBN moiety into a single molecular platform. The rationale behind this design was to further enhance binding avidity to αvβ3 by exploiting the multivalent effect, which increases local ligand concentration at the tumor site and facilitates receptor cross-linking. PET imaging studies in PC-3-bearing mice demonstrated significantly higher tumor accumulation and improved pharmacokinetics compared to monovalent and bivalent analogs. The addition of a second RGD motif did not compromise GRPR binding, thereby maintaining dual-receptor specificity while achieving improved retention and uptake kinetics [60].

Amraee et al. focused on chelator optimization, aiming to address the known in vivo instability of certain ^64^Cu-chelate complexes. They synthesized ^64^Cu-NODAGA-RGD-BBN, where NODAGA (1,4,7-triazacyclononane-1-glutaric acid-4,7-acetic acid) was employed to ensure higher kinetic and thermodynamic stability with ^64^Cu. In vitro receptor binding assays confirmed preserved dual-target affinity, while in vivo PET studies showed favorable tumor uptake, low background, and acceptable organ doses. These data highlight the relevance of chelator engineering in improving the pharmacological and imaging performance of BRL [61]. Durkan et al. introduced a structurally novel bispecific antagonist radiopharmaceutical, ^64^Cu-RGD-Glu-NO2A-6-Ahx-RM2, combining an RGD sequence with RM2, a GRPR antagonist with improved pharmacokinetics compared to agonists. Antagonist-based radioligands have shown advantages in terms of enhanced tumor penetration and reduced internalization-mediated degradation. In PC-3 tumor-bearing mice, the tracer exhibited high tumor uptake (2.13 ± 0.42% ID/g at 1 h p.i.) and favorable tumor-to-muscle and tumor-to-blood ratios. Competitive receptor-blocking experiments confirmed dual-receptor targeting. The study reinforces the growing interest in antagonist-based BRLs for imaging GRPR, where internalization is not a prerequisite for effective tumor visualization [62].

Most recently, Li et al. extended the application of dual-targeting radiotracers beyond GRPR to include the epidermal growth factor receptor (EGFR), developing [^64^Cu]NOTA-RGD-GE11. This construct combines the well-characterized RGD peptide with GE11, a non-mitogenic peptide ligand for EGFR, allowing for simultaneous targeting of integrins and EGFR, both of which are implicated in pancreatic cancer progression and resistance to therapy. In vivo PET imaging using a BxPC-3 pancreatic tumor model demonstrated robust tumor accumulation (2.95 ± 0.34% ID/g at 1 h p.i.), with high specificity validated by receptor-blocking studies. The design exemplifies the flexibility of hetero-dimeric strategies to accommodate different targeting vectors for tumor phenotypes with co-expression of clinically relevant biomarkers [63].

As mentioned for BRLs for SPECT use, to make a comparative analysis on the papers proposing BRLs for PET use is difficult to perform since all the above-mentioned studies were focused on different tumors, mainly breast cancer and PCa, and adopted different strategies. All the three PET isotopes that have been used for the labeling showed good in vitro and in vivo results and favorable dosimetry that foresee the implementation of PET-labeled BRLs into clinical practice. The choice would be based on the type of primary cancer and its metastases and should take into account the half-life of the PET isotope. As an example, ^64^Cu could be preferred to evaluate cerebral lesions or metastases at later time points given its longer half-life. However more pre-clinical and clinical studies on different cancer’s histotypes are still needed to definitely translate the use of labeled BRLs into clinical practice.

Table 2 provides an overview of the BRLs labeled with PET radioisotopes.

## 4. BRLs for Theranostic Use

Theranostics represents one of the most innovative and promising advancements in modern nuclear medicine, especially in oncology, where the ability to identify specific tumor targets enables not only for non-invasive diagnosis but also for targeted treatment and disease monitoring. In this context, hetero-bivalent radiopharmaceuticals could represent a new strategy thanks to their ability to bind multiple molecular targets, thereby compensating for potential tumor heterogeneity and paving the way toward increasingly personalized nuclear medicine.

### 4.1. Radiolabelling with Lutetium-177

Lutetium-177 is an ideal radionuclide for theranostic applications due to its favorable emission profile. It delivers effective β^−^ radiation (E_max_ ≈ 497 keV) for targeted radiotherapy, while simultaneously emitting γ photons at 113 keV and 208 keV, suitable for high-quality SPECT imaging. This dual functionality allows for precise tumor irradiation alongside real-time monitoring of radiopharmaceutical biodistribution.

PCa remains one of the main indications for theranostic approaches, and the heterogeneity in receptor expression in this tumor type has spurred the development of BRLs capable of targeting more than one biomarker simultaneously.

^177^Lu-DOTA-PSMA-Lys3-BBN heterodimer combines a PSMA inhibitor with a BBN analog for GRPR targeting. Synthesized through lysine residue conjugation and functionalized with DOTA, this compound demonstrated superior affinity compared to its monomeric counterparts. Biodistribution studies confirmed significantly increased tumor uptake and reduced off-target accumulation, indicating improved diagnostic contrast and therapeutic index [64]. Similarly, the PSMA-617/RM2 heterodimer, developed using copper-catalyzed azide-alkyne click chemistry (CuAAC) and radiolabeled with ^177^Lu or ^68^Ga, showed increased tumor uptake compared to the monomers. Despite relatively high renal uptake, the radiotracer showed faster blood clearance and improved T/B ratio, suggesting more favorable biodistribution than the individual components [42]. Other dual-targeting strategies have focused on integrin αvβ_3_, such as ^177^Lu-iPSMA-RGD. Although this compound showed lower binding affinity compared to its monomeric forms, it demonstrated in vitro inhibition of VEGFR2 phosphorylation, suggesting potential anti-angiogenic effects [65].

The heterodimer ^177^Lu-DOTA-RGD-BBN, targeting both αvβ_3_ and GRPR, showed good tumor uptake in PC-3 tumor models (5.88 ± 1.12% ID/g at 0.5 h), with decreasing retention over 48 h and favorable tumor-to-blood and T/M ratios. Specificity was confirmed through blocking studies, and dosimetric estimates support its potential use as a therapeutic approach [66]. A similar compound, the DOTA-RGD/RM2 heterodimer, incorporating flexible linkers (Glu and 6-Ahx), was labeled with ^177^Lu or 111In and tested in prostate PC-3 xenografts. The tracer achieved approximately 7.4% ID/g tumor uptake at 1 h, with substantial retention at 24 h (4.4% ID/g). Compared to monomers, it showed improved tumor accumulation and better clearance profiles, particularly with reduced hepatic uptake [23].

The heterodimer ^177^Lu-DOTA-DUPA-Ahx-BBN combines a PSMA ligand (DUPA, a glutamate analog) with a BBN antagonist for simultaneous targeting of PSMA and GRPR. The radiopharmaceutical demonstrated high affinity for both receptors (IC_50_ ≈ 3–6 nM), and in vivo studies in GRPR+/PSMA+ tumor-bearing mouse models showed high tumor uptake (~7.5% ID/g at 1 h) and low off-target accumulation, indicating an improved theranostic profile compared to the corresponding monomers [21].

In breast cancer, the co-expression of GRPR and folate receptors in various tumor subtypes has driven the development of radiopharmaceuticals targeting both receptors simultaneously. Among these, ^177^Lu-Folate-BBN combines a BBN analog with a folate moiety on a lysine-based scaffold. This DOTA-functionalized heterodimer showed high radiochemical purity (>98%) and serum stability for over 24 h. In vivo imaging studies in T47D tumor-bearing mice confirmed selective tumor localization and high absorbed radiation doses (23.9 ± 2.1 Gy at 74 MBq), with clear SPECT/CT visualization of tumor lesions. These findings support the potential of GRPR/FR-targeted heterodimers to enhance both specificity and therapeutic efficacy in FR+/GRPR+ breast cancers [67].

Together, these studies highly advocate the potential of BRLs to improve both the effectiveness and targeted ability of radionuclide-mediated theranostics. By targeting two or more tumor-associated receptors with homo-dimeric and combination-monomeric compounds, they enhance uptake and retention in tumors and can optimize tumor biodistribution with minimized off-target toxicities. Thus, they represent an important step forward in advancing personalized and tailored approaches to diagnosis and treatment of heterogeneous cancers, such as prostate and breast cancer.

### 4.2. Radiolabelling with Other Radioisotopes

Amongst the most commonly available isotopes for theranostics, some authors have selected radionuclides such as Yttrium-90, Iodine-131, and Rhenium-188. In particular, Yttrium-90 provides high-energy β^−^ emission, while Iodine-131 and Rhenium-188 combine therapy and imaging capabilities through their β^−^ and γ emissions.

An alternative chelation approach was applied to MAG2-RGD-BBN, labeled with ^188^Re for therapy in PCa. Despite moderate tumor uptake and high pancreas uptake rapidly decreasing over time, the compound showed low off-target retention and rapid renal clearance, but requires further optimization [68].

Finally, the heterodimer [RGD-Glu-(DO3A)-6-Ahx-RM2] labeled with ^86^Y/^90^Y exhibited high affinity for GRPR in PC-3 cells and rapid clearance from non-target tissue in tumor-bearing mouse. On the other hand, the radiopharmaceutical showed only moderate/low binding to αvβ_3_ in U87-MG cells. Tumor uptake and favorable biodistribution profiles support its theranostic potential [69].

In conclusion, the use of alternative radionuclides in the design of BRLs could further extend the theranostic repertoire. Each radionuclide presents advantages in terms of emission profile and constitutes a versatile tool for imaging and treatment depending on tumor type, receptor expression, and clinical purpose. The studies presented herein illustrate that different chelation approaches, from iodination to MAG2- and DO3A-based systems, were feasible and efficient for radiolabeling multivalent heterodimers directed towards relevant biomarkers (PSMA, GRPR and integrins). Although somatostatin receptor-targeting radiopharmaceuticals like [^125^I]I-BO530 exhibited outstanding tumor binding and retention, others like MAG2-RGD-BBN and ^86^Y/^90^Y-labeled heterodimers will require structural optimization for greater selectivity and decreased non-target tissue uptake. However, the promising biodistribution, low off-target accumulation, and receptor-specific uptake in preclinical models justify further investigation of these radionuclides for clinical translation.

**Figure 2:**
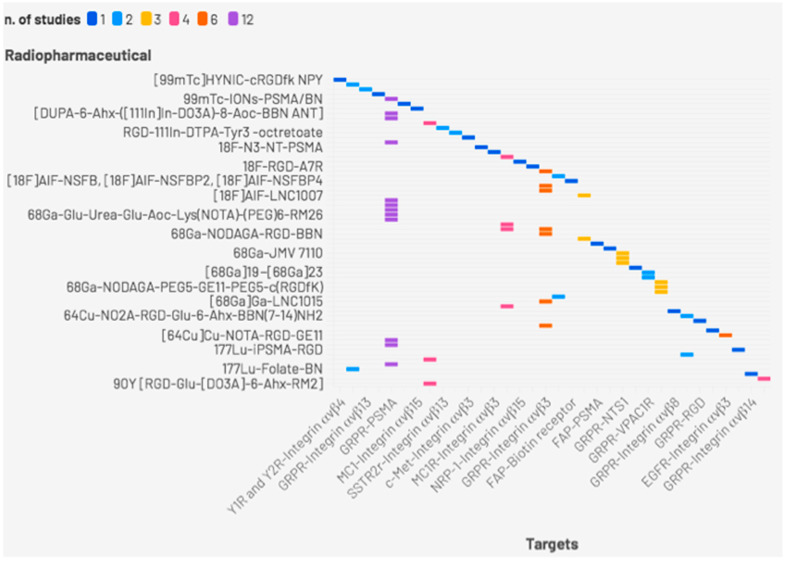
Graphical representation of papers published on labeled BRLs.

Table 3 summarizes the main features of BRLs mentioned above for theranostic use and shows a graphical representation of all the papers published on radiolabeled BRLs (Figure 2).

## 5. Conclusions and Future Perspectives

The application of BRLs has paved the way in molecular imaging and theranostic medicine, providing higher sensitivity, specificity, and contrast over the conventional mono-specific radioligands.

In this regard, the incorporation of BRLs in SPECT and PET has already demonstrated promising results for early lesion detection, TME analysis, and response treatment assessment. From a theranostic point of view, this represents a way to turn imaging into targeted therapy, with an increasing customization of their clinical strategies. However, several challenges are still open, such as synthesis and massive production, pharmacokinetic optimization, identification of the best target strategy, and clinical translation. In this optic, to better address these issues, new strategies and technologies that are emerging in recent years could be employed also in this expanding domain.

The increasing integration of artificial intelligence (AI) into scientific research, in particular, the applications of AI in drug design, machine learning, and generative models, have already shown promise in optimizing ligands–receptor interactions, predicting biodistribution profiles and accelerating the design of peptide-based radiotracers [70,71].

Synthesis procedures might also be optimized by using more advanced automated or semi-automated microwave synthesizers which employ more sophisticated techniques to improve synthetic performance, minimizing synthetic volumes and waste, thereby optimizing costs and reducing environmental impact [72].

Amongst these approaches, microfluidic platforms represent a novel technique for the automated synthesis of a large library of peptides in a microarray format, allowing rapid screening for interactions with molecular targets using biochemical or cell assays [73,74]. This technique would be an ideal tool for the optimization of BRLs production.

Finally, the development of trispecific radioligands would represent a significant advancement in targeting tumors that simultaneously overexpress multiple receptors. To date, only trispecific antibody-like proteins have been proposed for these purposes but also switchable ligands could be employed to further and better characterize TME heterogeneity [75].

A promising strategy to improve the pharmacokinetics of BRLs would be represented by the introduction of biodegradable linkers, such as enzyme-cleavable linkers that have been already successfully used to conjugate peptides and drugs for cancer therapy [76].

In conclusion, BRLs represent a strategic frontier in the future of nuclear medicine and will be essential tools for more accurate diagnoses and for more targeted and personalized treatments. Co-targeting FAP with integrin αvβ3 for imaging of CAFs and tumor angiogenesis simultaneously or RGD-BBN conjugates have been particularly explored and claim to enter soon in routine practice; however, further preclinical and clinical studies are needed to better assess the safety, the efficacy, and the clinical impact of radiolabeled BRLs. Aiming at improving cancer detection and management, multidisciplinary teams are also essential in promoting the implementation of novel, useful BRLs for personalized medicine.

## Figures and Tables

**Table 1:** Characteristics of BRLs radiolabeled with ^99m^Tc, ^111^In and ^125^I.

^99m^Tc										
BRL	Pathology	Peptide 1	Target 1	Peptide 2	Target 2	Chelator	Linker	Development Phase	Limitations	Ref.
**[^99m^Tc]HYNIC-cRGDfk NPY**	Breast cancer	NPY	Y1R and Y2R	cRDGfK	Integrin αvβ4	HYNIC-tricine-EDDA	8-amino-3,6-dioxaoctanoic acid	Pre-Clinical trial	High uptake in the stomach and intestines	[13]
**^99m^Tc-Bombesin-Folate**	Breast cancer	BBN	GRPR	Folate	FRα	HYNIC-tricine-TPPTS	Glu	Pre-Clinical trial	High renal and pancreatic uptake	[14]
**^99m^Tc-RGD-BBN**	Lung Carcinoma	BBN	GRPR	RGD	Integrin αvβ13	Cys(Acm)-Gly-Cys(Acm)	Gly-Gly-Cys-Gly	Pre-Clinical trial	Cancer model used was not dual-receptor	[18]
**^99m^Tc-RGD-BBN**	Breast cancer and axillary lymph nodes	BBN	GRPR	RGD	Integrin αvβ3	HYNIC-tricine-EDDA	Lys-Lys	Clinical trial	Low sensitivity for lesions with sizes less than 10 mm	[15]
**^99m^Tc-Tat-BN**	Different types of cancer	BBN	GRPR	Tat	Internalization	Glucoheptonate	Lys-Cys-Cys	Pre-Clinical trial	High uptake in kidneys and in non-target organs	[16]
**^99m^Tc-IONs-PSMA/BN**	Prostate cancer	BBN	GRPR	Lys-CO-Glu	PSMA	HYNIC-tricine-TPPTS	Glu	In vitro cell studies	Very preliminary data	[17]
**^99m^Tc-HYNIC-E-[c(RGDfk)-c(GX1)]**	Gliomas	GX1	Integrin α3β1	RGD	Integrin αvβ3	[Tc(H2O)3(CO)3]+	-	Pre-Clinical trial	Studied in a nonanatomical site for human tumors	[19]
**^99m^Tc-RGD-Lys-(Arg(11))CCMSH**	Melanoma	α-MSH	MC1	RGD	Integrin αvβ15	HYNIC-tricine-EDDA	-	Pre-Clinical trial	High renal uptake	[20]
**^111^In**										
**BRL**	**Pathology**	**Peptide 1**	**Target 1**	**Peptide 2**	**Target 2**	**Chelator**	**Linker**	**Development phase**	**Limitations**	**Ref.**
**[DUPA-6-Ahx-([^111^In]In-DO3A)-8-Aoc-BBN ANT]**	Prostate cancer	BBN	GRPR	DUPA	PSMA	DO3A	6-Ahx-8-Aoc	Pre-Clinical trial	High renal uptake	[21]
**^111^In-Glu-Urea-Glu-Aoc-Lys(NOTA)-(PEG)6-RM26**	Prostate cancer	RM26	GRPR	Glu-Urea-Glu-Aoc-Lys	PSMA	NOTA	Polyethylene glycol (PEG_6_)	Pre-Clinical trial	Rapid activity washout from tumors	[22]
** RGD-Glu-[^111^In-DO3A]-6-Ahx-RM2 **	Prostate cancer	RM2	GRPR	RGD	Integrin αvβ11	DOTA	Glu-6Ahx	Pre-Clinical trial	High pancreatic uptake	[23]
** RGD-^111^In-DTPA-octreotate **	Different types of cancer	Octreotate	Sst2r	RGD	Integrin αvβ13	DTPA	-	Pre-Clinical trial	High renal uptake	[24]
**RGD-^111^In-DTpA-Tyr3-octretoate**	Different types of cancer	Octreotate	Sst2r	RGD	Integrin αvβ14	DTPA	-	Pre-Clinical trial	High renal uptake	[25]
**^125^I**										
**BRL**	**Pathology**	**Peptide 1**	**Target 1**	**Peptide 2**	**Target 2**	**Chelator**	**Linker**	**Development phase**	**Limitations**	**Ref.**
**^125^I-cMBP-click-cRGDyk**	Gliomas	cMBP	c-Met	RGD	Integrin αvβ3	Chloramine-T	1 + 3 cyclo addition	Pre-Clinical trial	Low tumor uptake	[26]
** [^125^I]I-BO530 **	Prostate cancer	RM26	GRPR	PSMA-617	PSMA	IodoGen	Cu(I)-catalyzed cycloaddition	Pre-Clinical trial	High renal uptake	[27]

**Table 2:** Characteristics of BRLs radiolabeled with ^18^F, ^68^Ga, and ^64^Cu.

^18^F										
BRL	Pathology	Peptide 1	Target 1	Peptide 2	Target 2	Chelator	Linker	Development Phase	Limitations	Ref.
**^18^F-N3-NT-PSMA**	Prostate cancer	NT20.3	NTR1	Glu-urea-lys(Ahx)	PSMA	via azide–alkyne click reaction	-	Pre-Clinical trial	Low affinity to PSMA	[29]
**[^18^F]HBPL**	Melanoma	GG-Nle-c(DHfRWK)	MC1R	(c(RGDfK)	Integrin αvβ3	SiFAlin	Different linkers	Pre-Clinical trial	Low radiochemical yield	[28]
**^18^F-FB-PEG_3_-RGD-BBN **	Breast cancer	BBN	GRPR	RGD	Integrin αvβ4	-SFB	PEG3	Pre-Clinical trial	Rapid wash-out	[30]
**^18^** **FAl-NOTA-RGD-ATWLPPR**	Gliomas	ATWLPPR	NRP-1	c(RGDyK)	Integrin αvβ15	-SFB	glutamic acid.	Pre-Clinical trial	low receptor-binding affinity	[31]
**^18^** **F-RGD-A7R**	Different types of cancer	ATWLPPR A7R	VEGFR	RGD	Integrin αvβ14	-SFB	glutamic acid.	Pre-Clinical trial	Unfavorable uptakes in stomach and intestine	[32]
**^18^F-RGD-BBN**	Different types of cancer	BBN	GRPR	RGD	Integrin αvβ3	-SFB	glutamic acid.	Pre-Clinical trial	Low radiochemical yield	[33]
**^18^** **F-T2P**	Prostate cancer	TJ12P2	Glypican-3 (GPC3)	2-PMPA	PSMA	NOTA	PEG	Pre-Clinical trial	Short half-life	[34]
**[^18^F]AlF-NSFB, [^18^F]AlF-NSFBP_2_, [^18^F]AlF-NSFBP_4_**	Different types of cancer	Onco-FAP	FAP	Biotin	Biotin receptor	DFO	PEG-MAL	Pre-Clinical trial	Necessity of rational design and pharmacomodulation	[35]
**^18^F-FB-PEG(3)-Glu-RGD-BBN**	Different types of cancer	BBN	GRPR	RGD	Integrin αvβ3	EG3 spacer	glutamate linker	Pre-Clinical trial	Prominent uptake in kidneys at early time points	[36]
**^18^F-FB-AEADP-BBN-RGD**	Prostate cancer	BBN	GRPR	RGD	Integrin αvβ3	-SFB	glutamate linker	Pre-Clinical trial	Low specificity	[37]
**[^18^F]AlF-LNC1007**	Different types of cancer	FAP-2286	FAP	RGD	Integrin αvβ3	NOTA	-	Clinical trial	Fast clearance and short retention time	[38]
**^64^** **Ga**										
**BRL**	**Pathology**	**Peptide 1**	**Target 1**	**Peptide 2**	**Target 2**	**Chelator**	**Linker**	**Development phase**	**Limitations**	**Ref.**
**^68^** **Ga-Glu-urea-Lys-HBED-CC-BZH_3_**	Prostate cancer	BZH3	GRPR	Glu-urea-Lys(Ahx)-HBED-CC	PSMA	HBED-CC		Pre-Clinical trial	High kidney and pancreatic uptake	[39]
**^68^Ga-HE2**	Prostate cancer	H2N-PEG2-BN(6–14)	GRPR	Glu-ureido-Lys	PSMA	HBED-CC		Pre-Clinical trial	Tracer accumulation in nontarget organs	[40]
**^68^** **Ga-iPSMA-BN**	Prostate cancer	Lys3-BBN	GRPR	Nal-Lys-CO-Glu-OH	PSMA	DOTA	GMBS	Pre-Clinical trial	High renal uptake	[41]
**^68^Ga-Glu-Urea-Glu-Aoc-Lys(NOTA)-(PEG)_6_-RM26 **	Prostate cancer	RM26	GRPR	Glu-Urea-Glu-Aoc-Lys	PSMA	NOTA	PEG	Pre-Clinical trial	Low tumor-to-nontumor ratios	[22]
**^68^** **Ga-PSMa-617/RM2**	Prostate cancer	RM2	GRPR	PSMA-617	PSMA	NOTA	PEG/Aoc-Phe	Pre-Clinical trial	Pharmacokinetics to be improved	[42]
**^68^Ga-FB-PEG3-RGD-BBN **	Breast cancer	BBN	GRPR	RGD	Integrin αvβ4	-SFB	PEG3	Pre-Clinical trial	Low affinity to GPPR	[30]
**^68^Ga-BBN-RGD **	Prostate cancer	BBN	GRPR	RGD	Integrin αvβ4	NOTA	-	Clinical trial	Expression status in metastases unknown	[44]
**^68^** **Ga-BBN-RGD**	Breast cancer	BBN	GRPR	RGD	Integrin αvβ3	NOTA	glutamic acid.	Clinical trial	False-positive cases	[45]
**^68^** **Ga-NODAGA-RGD-BBN**	Prostate cancer	BBN	GRPR	RGD	Integrin αvβ3	NODAGA	-	Pre-Clinical trial	High renal uptake	[46]
**^68^Ga-FAPI-RGD **	Different types of cancer	FAPI-02	FAP	RGD	Integrin αvβ3	NOTA	PEG	Clinical trial	Limited number of patients and no healthy subjects involved	[47]
**^68^Ga-FAPI-RGD **	Different types of cancer	FAPI-02	FAP	RGD	Integrin αvβ3	NOTA	PEG	Clinical trial	High uptake in thyroid and pancreas	[48]
**[^68^Ga]Ga-PSFA-01**	Prostate cancer	FAPI-04	FAP	EuK (PSMA11)	PSMA	HBED-CC		Clinical trial	Only one patient studied	[49]
**^68^** **Ga-FAPI-LM3**	Pulmonary fibrosis	FAPI-46	FAP	LM3	SSTR2	-		Pre-Clinical trial	The mice model do not replicate the human pathology	[50]
**^68^** **Ga-JMV 7110**	Breast cancer	BBN analogs	GRPR	NT analogs	NTS1	DOTA	βAla	In vitro cell studies	Loss of NTS1-specific internalization	[51]
**^68^** **Ga-JMV 7253**										
**^64^** **Ga-JMV 7266**									No specific binding	
**^68^Ga-HBPL**	Breast cancer	BBN_7–14_	GRPR	[Lys^4^, Trp^5^, Nle^7^] BVD_15_	NPY (Y_1_)R	NODAGA	PEG	Pre-Clinical trial	High kidney and liver uptake	[52]
**[^68^Ga]19–[^68^Ga]23**	Different types of cancer	Aminooxy-PESIN	GRPR	Aminooxy-TP3805	VPAC 1R	NODAGA	PEG	In vitro evaluation	High renal uptake	[53]
**[^68^Ga]Ga-9**	Prostate cancer	PESIN	GRPR	PACAP-27	VPAC 1R	NODAGA	(PEG)3	Pre-Clinical trial	Suboptimal pharmacokinetic	[54]
**^68^** **Ga-NODAGA-PEG_3_-GE_11_-PEG_3_-c(RGDyK)**	(NODAGA-PEG 3-GE11-PEG3-c(RGDyK)	GE11	EFGR	RGD	Integrin αvβ3	NODAGA	(PEG)3	Pre-Clinical trial	Low affinity to EGFR	[55]
**^68^Ga-NODAGA-PEG_5_-GE_11_-PEG_5_-c(RGDfK)**	NODA GA-PEG5-GE11-PEG5-c(RGDfK)						(PEG)5			
**^68^Ga-NOTA-RGD-cys-6-Ahx-GE11**	(68)Ga-NOTA-RGD-cys-6-Ahx-GE11	GE11	EFGR	RGD	Integrin αvβ3	NOTA	6-aminohexanoic	In vitro evaluation	Very preliminary data	[56]
**^68^Ga-T2P**	Prostate cancer	TJ12P2	Glypican-3 (GPC3)	2-PMPA	PSMA	NOTA	PEG	Pre-Clinical trial	Notable uptake in liver and kidneys	[35]
**[^68^Ga]Ga-LNC1015**	Different types of cancer	RM26	GRPR	RGD	Integrin αvβ3	NOTA	n.d.a.	Clinical trial	Rapid tumor wash out and high kidney uptake	[57]
**^64^** **Cu**										
**BRL**	**Pathology**	**Peptide 1**	**Target 1**	**Peptide 2**	**Target 2**	**Chelator**	**Linker**	**Development phase**	**Limitations**	**Ref.**
**^64^Cu-FB-PEG3-RGD-BBN **	Breast cancer	BBN	GRPR	RGD	Integrin αvβ4	-SFB	PEG3	Pre-Clinical trial	High and prolonged liver uptake	[31]
**^64^Cu-NOTA-RGD-bombesin**	Different types of cancer	BBN	GRPR	RGD	Integrin αvβ8	NOTA	glutamic acid.	Pre-Clinical trial	Significant renal and abdominal uptake	[58]
**^64^Cu-NO2A-RGD-Glu-6-Ahx-BBN(7-14)NH2**	Prostate cancer	BBN(7-14)NH(2)	GRPR	RGD	Integrin αvβ9	NO2A	Glu-6-Ahx	Pre-Clinical trial	High pancreatic uptake	[59]
** E[c(RGDyK)]2-PEG3-Glu-(Pro-Gly)12-BBN(7-14)-NH2 (RGD2-PG12-BBN) **	Prostate cancer	BBN	GRPR	RGD	RGD	NODAGA	[Pro-Gly]x	Pre-Clinical trial	Low tumor uptake	[60]
** [^64^Cu]Cu-NODAGA-RGD-BBN **	GRPR+ tumors	BBN	GRPR	RGD	Integrin αvβ3	NODAGA	-	Pre-Clinical trial	Uptake in GRPR-expressing organs	[61]
**[RGD-Glu-[^64^Cu-NO2A]-6-Ahx-RM2]**	Prostate cancer	RM2	GRPR	RGD	Integrin αvβ10	NOTA	glutamic acid.	Pre-Clinical trial	High pancreatic and hepatic uptake	[62]
**[^64^Cu]Cu-NOTA-RGD-GE11**	pancreatic cancer	GE11	EGFR	RGD	Integrin αvβ3	NOTA	PEG4	Pre-Clinical trial	High renal uptake	[63]

**Table 3:** Characteristics of BRLs radiolabeled with ^177^Lu, ^188^Re, ^89/90^Y for theranostic use.

^177^Lu										
BRL	Pathology	Peptide 1	Target 1	Peptide 2	Target 2	Chelator	Linker	Development Phase	Limitations	Ref.
**^177^Lu-DOTA-iPSMA-Lys^3^-BN **	Prostate cancer	Lys3-BBN	GRPR	Nal-Lys-CO-Glu-OH	PSMA	DOTA	-	In vitro evaluation	High pancreatic uptake	[64]
**^177^** **Lu-PSMA-617/RM2**	Prostate cancer	RM2	GRPR	PSMA-617	PSMA	NOTA	PEG/Aoc-Phe	In vitro evaluation	Tumor/pancreas ratio increased over time	[43]
**^177^Lu-iPSMA-RGD**	Prostate cancer	iPSMA	PSMA	RGD	Integrin αvβ13	DOTA	-	Pre-Clinical trial	Very preliminary data	[65]
**^177^Lu-DO3A-RGD-BBN**	Prostate cancer	BBN	GRPR	RGD	Integrin αvβ9	DO3A	6-Ahx	Pre-Clinical trial	High pancreatic uptake	[66]
** RGD-Glu-[^177^Lu-DO3A]-6-Ahx-RM2 **	Prostate cancer	RM2	GRPR	RGD	Integrin αvβ11	DO3A	Glu-6Ahx	Pre-Clinical trial	Lower efficacy than monovalent RM2 antagonists	[24]
**[DUPA-6-Ahx-([^177^Lu]In-DO3A)-8-Aoc-BBN ANT]**	Prostate cancer	BBN	GRPR	DUPA	PSMA	DO3A	6-Ahx-8-Aoc	Pre-Clinical trial	High uptake in lung, liver, kidney, and spleen	[22]
**^177^Lu-Folate-BN **	Breast cancer	BBN	GRPR	Folate	FRα	DOTA	-	Pre-Clinical trial	High renal uptake	[67]
**^188^** **Re**										
**BRL**	**Pathology**	**Peptide 1**	**Target 1**	**Peptide 2**	**Target 2**	**Chelator**	**Linker**	**Development phase**	**Limitations**	**Ref.**
**^188^Re-MAG2-RGD-BBN**	Prostate cancer	BBN	GRPR	RGD	Integrin αvβ14	MAG2	glutamate linker	Pre-Clinical trial	Low specific activity	[68]
**^89^** **Y/** **^90^** **Y**										
**BRL**	**Pathology**	**Peptide 1**	**Target 1**	**Peptide 2**	**Target 2**	**Chelator**	**Linker**	**Development phase**	**Limitations**	**Ref.**
**^86^YRGD-Glu-[DO3A]-6-Ahx-RM2] **	Prostate cancer	RM2	GRPR	RGD	Integrin αvβ11	DO3A	-	Pre-Clinical trial	Moderate affinity for αvβ3	[69]
**^90^Y [RGD-Glu-[DO3A]-6-Ahx-RM2] **										

## Data Availability

Not applicable.
